# Anxiety and depression in patients with suspected carpal tunnel syndrome – A case controlled study

**DOI:** 10.1002/brb3.1342

**Published:** 2019-06-17

**Authors:** Lucy Moira McCallum, Nicholas Anthony Damms, Ptolemaios Georgios Sarrigiannis, Panagiotis Zis

**Affiliations:** ^1^ Sheffield Institute for Translational Neuroscience Sheffield UK; ^2^ Academic Department of Neurosciences Sheffield Teaching Hospitals, NHS Trust Sheffield UK; ^3^ Medical School University of Cyprus Nicosia Cyprus

**Keywords:** anxiety, carpal tunnel syndrome, depression, neurophysiology, pain

## Abstract

**Purpose:**

Carpal tunnel syndrome (CTS) is a common entrapment neuropathy causing significant, and often disabling, pain. We aimed to establish the prevalence of anxiety and depressive symptoms in patients who were referred with suspected CTS and identify potential determinants.

**Methods:**

All patients underwent nerve conduction studies (NCS) and were classified into mild, moderate, severe, and no CTS groups. Volunteers, without symptoms or signs of CTS, formed the control group. Anxiety and depressive symptoms were assessed via the Hospital Anxiety and Depression Scale.

**Results:**

Ninety‐one patients and 41 controls were recruited. Following NCS the patients were classified as follows: mild CTS (*n* = 20), moderate CTS (*n* = 21), severe CTS (*n* = 11), and no CTS (*n* = 31). CTS patients had significantly higher depression scores compared to controls but not anxiety scores. Patients experiencing pain and itchiness had significantly higher anxiety scores compared to those who did not. Patients who reported symptoms suggestive of CTS but did not meet the electrodiagnostic criteria for a diagnosis had significantly higher anxiety and depression scores compared to CTS patients and controls.

**Conclusions:**

Patients suffering with CTS may be at an increased risk of depression. Experiencing pain in CTS may further increase the likelihood of experiencing mental health difficulties. Poor mental health can give rise to functional symptoms, similar to those seen in CTS, demonstrating the need for electrophysiological testing before considering surgical intervention.

## 1 INTRODUCTION

Carpal tunnel syndrome (CTS) is a condition caused by compression of the median nerve within the carpal tunnel at the wrist. It is well regarded as the most common nerve entrapment syndrome (Atroshi et al., 1999). It is most prevalent between the ages of 40 and 60 years; however, it can affect people of all ages (Patijn et al., 2011).

Symptoms of CTS commonly include numbness, tingling, pain, muscle cramps, and burning sensations. These normally affect the thumb, index, middle, and lateral half of the ring finger. Symptoms are common during the night, with patients often reporting being woken by tingling and pain in the affected hand (Aroori & Spence, 2008). As the condition progresses reduced grip strength becomes evident (Alfonso, Jann, Massa, & Torreggiani, 2010). In advanced cases, wasting of the thenar muscles may occur (Patijn et al., 2011).

Due to these symptoms, patients may be prevented from executing tasks crucial to their work, impacting on their ability to continue in employment. In addition, chronic pain may have a substantial negative impact on a patient's life. Several existing studies appear to suggest that the rate of anxiety and depression in CTS patients is significantly higher than that observed in the general population (Beleckas et al., 2018; Moghadam‐Ahmadi, Bidaki, Shahriari Sarhadi, Vakilian, & Sharifi, 2017).

As in all conditions causing chronic pain, CTS has a negative effect on mental health in patients (Zis et al., 2017). The presence of chronic pain in CTS may play a large role in the mental health difficulties seen in CTS patients.

The aim of this study was to establish the prevalence of anxiety and depression symptoms in patients who were referred with suspected CTS and identify potential determinants.

## 2 MATERIALS AND METHODS

### 2.1 Participants

Participants displaying symptoms suggestive of CTS were recruited from CTS clinics. The exclusion criteria comprised: any person under the age of 18, those lacking capacity to provide written informed consent, those who also had symptoms suggestive of ulnar neuropathy, which was confirmed electrophysiologically, and those who had undergone previous CTS release surgery. Volunteers, with no history of previous or current nerve entrapments or conditions causing chronic pain, were recruited as controls.

All participants gave their full written informed consent before taking part in the study and they were not paid to partake.

### 2.2 Demographics and clinical evaluation

Demographic data included gender and age. Clinical information included comorbidity with diabetes, cancer, and weekly alcohol consumption. In the United Kingdom, current recommended upper permitted limit is 21 units of alcohol weekly (3 units daily).

Symptom details were also obtained and included type of symptom (numbness, tingling, pins and needles, itchiness, burning, muscle cramps, and pain), duration of pain when present, and intensity of pain on a Visual Analogue Scale (ranging from 0 to 10).

Edinburgh Handedness Inventory (EHI) (Oldfield, 1971) was used to assess handedness in participants. The results from the EHI were used to determine whether the CTS was in the patients' dominant or non‐dominant hand.

The Hospital Anxiety and Depression scale (HADS; Zigmond & Snaith, 1983) was used to assess depression and anxiety symptoms. This is a validated 14‐item tool that generates two scores (ranging from 0 to 21): an anxiety subscale (HADS‐A) and a depression subscale (HADS‐D). Each subscale consists of seven questions, which are scored on a 4‐point scale (ranging from 0 to 3).

### 2.3 Neurophysiological assessment

The following parameters measured using Natus equipment were used for this study:

1. Orthodromic Median sensory nerve action potential (SNAP). The active recording ring electrode was placed around the base of the proximal phalanx of the third digit. The reference ring electrode was placed between the proximal phalanx and the middle phalanx of the third digit. Stimulation was 2 cm proximal to the wrist crease.
2. Orthodromic Ulnar SNAP. The active recording ring electrode was placed around the base of the proximal phalanx of the fifth digit. The reference ring electrode was placed between the proximal phalanx and the middle phalanx of the fifth digit. Stimulation was 2 cm proximal to the wrist crease.
3. Median compound muscle action potential (CMAP). The active recording electrode was placed over the belly of the abductor pollicis brevis (APB). The reference recording electrode was placed over the tendon of the APB at the base of the proximal phalanx of the thumb. Stimulation was 2 cm proximal to the wrist crease.

### 2.4 Classification of CTS

The classification of mild, moderate, and severe CTS was adapted from the Canterbury grading scale for CTS as follows (Bland, 2000):

1. *Mild CTS*: median motor distal latency (MDL) < 4.5 ms, median sensory conduction velocity (SCV) < 45 m/s.
2. *Moderate CTS*: median MDL ≥ 4.5 ms, median SNAP > 0 μV.
3. *Severe CTS*: median MDL ≥ 4.5, absent median SNAP.
4. *No CTS*: median MDL < 4.5 ms, median SCV ≥ 45 m/s.

For patients who had bilateral CTS with a different CTS classification in each hand, the worst severity they were given was taken as their overall CTS classification.

### 2.5 Statistical analyses

Statistical analyses were performed using the Statistical Package for the Social Sciences (SPSS, version 25) for windows. A Chi‐squared test was used to identify whether frequencies of clinical characteristics and demographics differed significantly between groups.

The data failed to meet the assumption of normally distributed data assessed by the Shapiro‐Wilk test. Therefore, the means of continuous variables between two or more groups were compared using the Mann‐Whitney *U* test or the Kruskal‐Wallis one‐way analysis of variance test, respectively. The Bonferroni correction was applied for multiple comparisons.

Correlation analyses, using Spearman's test, were performed to assess the relationship between continuous variables.

A *p* < 0.05 was considered to be statistically significant.

## 3 RESULTS

### 3.1 Study population

Ninety‐one patients with symptoms suggestive of CTS and 41 controls were recruited. Based on the results from their NCS, the 91 patients were classified into mild CTS (*n* = 20), moderate CTS (*n* = 21), severe CTS (*n* = 11), and “no CTS” (*n* = 39).

Itchiness was more prevalent in mild CTS (60%), compared to moderate (28.6%) or severe (18.2%). No other significant differences regarding neuropathic symptoms were observed between the groups. Demographic and clinical characteristics for all groups are summarized in Table 1.

**Table 1:** Demographic and clinical characteristics of the subjects included in this study

	No CTS (*N* = 39)	Mild CTS (*N* = 20)	Moderate CTS (*N* = 21)	Severe CTS (*N* = 11)	Controls (*N* = 41)	*p* value
Demographics						
Age, in years (*SD*)	45.7 (12.7)	56.3 (13.0)	60.6 (10.5)	68.91 (7.9)	47.12 (15.6)	<0.001
Male (%)	18 (46.2)	4 (20.0)	7 (33.3)	4 (36.4)	14 (34.1)	0.392
Clinical characteristics						
Exceeding permitted alcohol limit (%)	8 (20.5)	4 (20.0)	3 (14.3)	2 (18.2)	13 (31.7)	0.548
History of diabetes (%)	5 (12.8)	0 (0.0)	5 (23.8)	2 (18.2)	0 (0)	0.010
History of cancer (%)	0 (0.0)	0 (0.0)	2 (9.5)	2 (18.2)	0 (0)	0.005
Duration of symptoms, in months (*SD*)	30.4 (33.8)	19.7 (17.2)	37.5 (64.7)	13.6 (12.5)	*N*/A	0.477
Bilateral CTS	*N*/A	9 (45.0)	11 (52.4)	8 (72.7)		
Unilateral CTS		11 (55.0)	10 (47.6)	3 (27.3)	*N*/A	0.329
Numbness	35 (89.7)	17 (85)	21 (100)	10 (90.9)	*N*/A	0.378
Tingling	37 (94.8)	19 (95)	21 (100)	11 (100)	*N*/A	0.641
Pins and Needles	36 (92.3)	17 (85)	19 (90.5)	11 (100)	*N*/A	0.553
Itchiness	16 (41)	12 (60)	6 (28.6)	2 (18.2)	*N*/A	0.082
Burning	14 (35.9)	10 (50)	8 (38.1)	4 (36.4)	*N*/A	0.754
Muscle cramps	23 (59)	10 (50)	9 (42.9)	4 (36.4)	*N*/A	0.477
Pain	32 (82.1)	18 (80)	16 (76.2)	9 (81.8)	*N*/A	0.957
Pain intensity (*SD*)	7.03 (3.44)	6.85 (3.72)	6.86 (4.08)	6.36 (4.10)	*N*/A	0.962

Note

CTS, Carpal tunnel syndrome.

### 3.2 Symptoms of anxiety

The “No CTS” group had the highest mean HADS‐A score (9.4 ± 4.1), followed by the severe CTS (7.0 ± 5.5), mild CTS (6.8 ± 4.5), moderate CTS (6.5 ± 3.5), and control group (5.4 ± 3.3). Post hoc comparisons showed that HADS‐A scores were only significantly higher in the “No CTS” group compared to the healthy controls (*P* < 0.001). No significant differences occurred between any other groups.

### 3.3 Symptoms of depression

The “No CTS” group had the highest mean HADS‐D score (6.9 ± 4.0), followed by the severe CTS (5.6 ± 3.8), moderate CTS (5.2 ± 2.8), mild CTS (4.5 ± 3.1), and control group (2.4 ± 2.1). Post hoc comparisons showed that HADS‐D scores were significantly higher in the moderate CTS, severe CTS, and “No CTS” groups compared to the control participants. No significant differences occurred between any other groups.

### 3.4 The role of laterality and dominance

No statistically significant differences were found between bilateral CTS participants (*N* = 28) and unilateral CTS patients (*N* = 24) on HADS‐A and HADS‐D scores. Moreover, within the unilateral CTS participants there were no statistically significant differences on HADS‐A and HADS‐D scores between those with CTS in their dominant hand (*N* = 17) and those with CTS in their nondominant hand (*N* = 6).

### 3.5 The role of pain

Pain was reported by 75 patients (82.4%). HADS‐A test scores were significantly higher in those who reported pain compared to those who reported no pain (8.3 ± 4.5 vs. 6.0 ± 3.6, *p* = 0.042). There was no significant correlation found between the pain intensity and the HADS‐A score (Spearman's rho 0.129, *p* > 0.05). HADS‐D scores did not vary significantly between participants who reported pain and those who reported no pain.

### 3.6 The role of itchiness

Itchiness was reported by 36 patients (39.6%). The HADS‐A and HADS‐D scores were significantly higher in those who reported itchiness compared to those who did not (9.3 ± 4.7 vs. 6.9 ± 4.0, *p* = 0.013 and 6.7 ± 3.8 vs. 5.2 ± 3.3, *p* = 0.048, respectively).

## 4 DISCUSSION

In this present study, we investigated the presence of symptoms of anxiety and depression in patients with symptoms suggestive of CTS. We found that symptoms of depression are higher in patients with moderate and severe CTS, as well as patients within the “no CTS” group. Although anxiety was not more prevalent in the CTS group, patients who were complaining of CTS symptoms, which was not confirmed electrophysiologically, had higher HADS‐A scores.

Our findings are similar to those reported by Emir et al. (2013) who found that pregnant women with CTS had significantly higher depression but not anxiety scores compared to healthy controls. However, Beleckas et al. (2018) and Moghadam‐Ahmadi et al. (2017) reported that both the depression and the anxiety rates are significantly higher in CTS patients compared to general population.

A novelty of our study was that, we included patients who had symptoms suggestive of CTS that was not eventually confirmed electrophysiologically. Such patients showed higher anxiety and depression scores compared to the controls. A possible explanation for this is that anxiety about health might enhance symptom perception. Interestingly, in the no CTS group, a number of patients had history of diabetes, which can cause small fiber neuropathy (Lacomis, 2002), a significantly painful condition. Therefore, it is possible that the symptoms reported by patients classified as “no CTS” were secondary to small fiber neuropathy.

Moreover, patients with poor mental health, especially anxiety, are more likely to seek medical attention, exaggerate symptoms, and have lower resilience for dealing with illness (Cameron, Leventhal, & Love, 1998). Hazard et al. (Hazard, Bendix, & Fenwick, 1991) demonstrated that patients with poor mental health self**‐**reported worse disability levels than those with no mental health difficulties, despite an objective measure of physical functioning showing no difference between participants. Furthermore, Howren and Suls (Howren & Suls, 2011) identified that inducing anxiety in patients meant they were more likely to report physical symptoms than non‐anxious patients. Thus, mental health difficulties, especially anxiety, can cause patients to experience physical symptoms that are not attributed to any physical cause. This is an important factor to consider when offering therapeutic treatment for CTS, including CTS release surgery. Clearly, a clinical assessment of CTS per se is not sufficient. Neurophysiological evaluation is an important part of the CTS diagnostic process and should always be conducted prior to CTS treatment to avoid treating those who report CTS symptoms but whose median nerve conduction parameters are normal.

Our study demonstrated a link between anxiety levels and the presence of pain. This is supported by previous research investigating the prevalence of anxiety in patients with chronic pain (Katon & Sullivan, 1990; Kroenke et al., 2013). In our study population, however, we found no significant correlations between pain ratings and anxiety test scores, suggesting it was the presence of pain and not the severity of the pain that influenced participants' anxiety scores.

Apart from pain, our study demonstrated that patients experiencing itchiness presented with poorer mental health. An interesting finding was that itchiness appeared to be more prevalent in the mild CTS group compared to the severe group. This could suggest that itchiness as a symptom is more prevalent earlier on in the disease course or that this symptom is noticed less as time goes on. Currently, there is little published research investigating symptom changes during the disease course of CTS. As itchiness appears to be reported earlier in the disease course, this symptom should be given adequate attention clinically as identifying CTS early can reduce the likelihood of needing CTS release surgery.

In conclusion, patients with CTS have an increased risk of depressive and anxiety symptoms. Poor mental health, especially anxiety, can enhance symptom perception, or even cause functional symptoms that may present like CTS, hence neurophysiology testing is important before considering therapeutic treatment for CTS.

## CONFLICTS OF INTEREST

None.

## ETHICAL APPROVAL

This study was granted ethical approval by the NRES committee West Midlands‐Coventry & Warwickshire (REC number: 18/WM/0095). All procedures performed were in accordance with the ethical standards of the institutional and/or national research committee and with the 1964 Helsinki declaration and its later amendments or comparable ethical standards.

## Data Availability

The data that support the findings of this study are available from the corresponding author upon reasonable request.
